# Sonication Fluid Isolation of Peptoniphilus asaccharolyticus After Total Hip Arthroplasty

**DOI:** 10.7759/cureus.21419

**Published:** 2022-01-19

**Authors:** Michail Sarantis, Chrisa Argyrou, Dimitrios Tzefronis, Sophia Stasi, George Macheras

**Affiliations:** 1 4th Orthopaedic Department, KAT General Hospital, Athens, GRC; 2 Physiotherapy Department, Laboratory of Neuromuscular and Cardiovascular Study of Motion, University of West Attica, Attica, Greece, Athens, GRC

**Keywords:** two stage revision, anaerobe, periprosthetic joint infection, sonication, peptoniphilus asaccharolyticus

## Abstract

*Peptoniphilus asaccharolyticus *is a gram-positive anaerobic coccus found on the skin, vagina, and gut, where it acts as an opportunistic pathogen or as part of polymicrobial infections of chronic wounds or diabetic ulcers*. *We present a case of a 68-year-old woman who was diagnosed with a late prosthetic hip arthroplasty infection caused by *P. asaccharolyticus* and isolated from sonication fluid cultures. Despite the fact that evidence is scarce, its role and pathogenicity in more severe infections should not be underestimated.

## Introduction

Periprosthetic joint infection (PJI) is a common and devastating complication of total hip arthroplasties, occurring at a rate of 1-2% [1]. Although anaerobic microorganisms count for 3% to 6% of PJI [1], they lead to serious complications, morbidity, poor outcomes, and increased healthcare costs in the same way as common causative pathogens. Gram-positive anaerobic cocci (GPAC)-usually identified in infections caused by several types of microorganisms-represent around 30% of anaerobic microorganisms isolated from tissue samples. Routinely, GPACs are not fully identified due to difficulties in culture, identification, and isolation. *Peptoniphilus asaccharolyticus* is a GPAC, usually part of the vaginal and gut microbiota [2]. Mostly, it acts occasionally as part of polymicrobial infections and is often present in chronic wounds and diabetic ulcers [2]. However, there is not enough evidence on the exact contribution of *P. asaccharolyticus* to more devastating conditions like periprosthetic joint infections. We present a case of periprosthetic joint infection due to *P. asaccharolyticus* (mono-infection) and aim to raise awareness about this underestimated pathogen.

## Case presentation

A 68-year-old woman who had a left total hip arthroplasty (THA) four years ago, presented to our emergency department with mild but persistent pain in the groin and a progressive inability to bear weight in the left hip over the last six weeks. She was a non-drinker/non-smoker and was under treatment for hypothyroidism, dyslipidemia, and depression. She reported a pain-free and motion-free period of three and a half years after THA.

Upon admission, her temperature was 36.8 °C, her blood pressure was 127/83 mmHg, and her heart rate was 76 bpm. She weighed 81 kg and her body mass index was 29.1 kg/m^2^. She denied any bladder or bowel symptoms before. The skin at the surgical site was clear without any sign of infection. During the physical examination, the previous well-functioning hip joint was stiff and painful, with a restricted range of motion, especially with rotation. The patient was not able to do full weight-bearing and used crutches.

**Figure 1:**
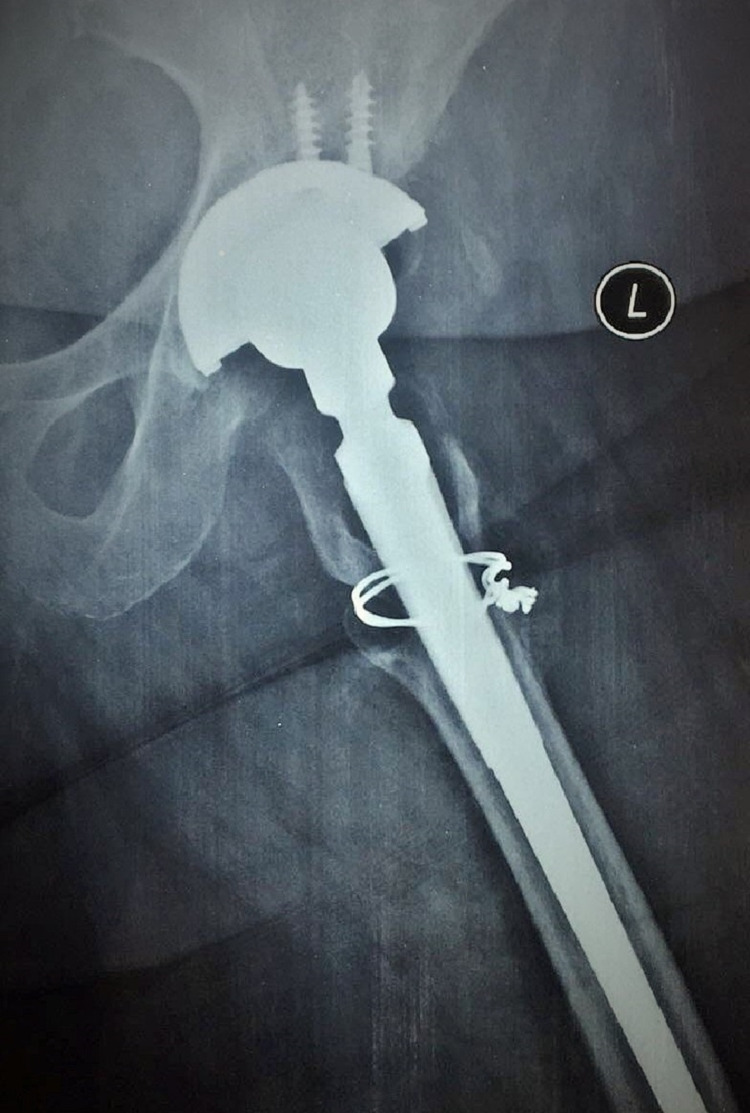
Preoperative radiographic evaluation

Routine blood test results and serum inflammation markers were evaluated and described as shown in Table 1. Her blood glucose and thyroid hormones were within normal values. Blood and urine cultures were negative. The radiograph evaluation showed osteolysis of the proximal metaphyseal femoral part, especially in zones 1, 7, and 8 according to the Gruen classification system (Figure 1). Hip aspiration under CT guidance was performed and *P. assacharolyticus* was isolated.

**Table 1:** Laboratory results WBC: white blood cell, HgB: haemoglobin, PLT: platelets, ESR: erythrocyte sedimentation rate, CRP: C-reactive protein

Laboratory blood results	
WBC	8.651/μL (normal 4.600–10.200)
HgB	12.1 mg/dL (normal: 12–18)
PLT	351.000/μL (normal: 130.000–400.000)
ESR	119 mm/h (normal: 0–20)
CRP	10.2 mg/dL (normal: <0.5)

The patient was diagnosed with a late periprosthetic infection and it was decided to undergo a two-stage revision hip arthroplasty. The procedure was performed under spinal anesthesia and a standard posterior hip approach was performed. Tissue samples and joint fluid were taken for cultures and histology (six samples were taken from sites with obvious inflammatory changes). All necrotic soft tissues were debrided. Infected prostheses were removed and sent for sonication. We used the explant device to remove the acetabular prosthesis. Extended trochanteric osteotomy was performed for the removal of the femoral prosthesis. Thorough washing and lavage took place. A static antibiotic spacer containing tobramycin and vancomycin was used and started immediately intravenously with vancomycin until the microbiology results were obtained.

**Figure 2:**
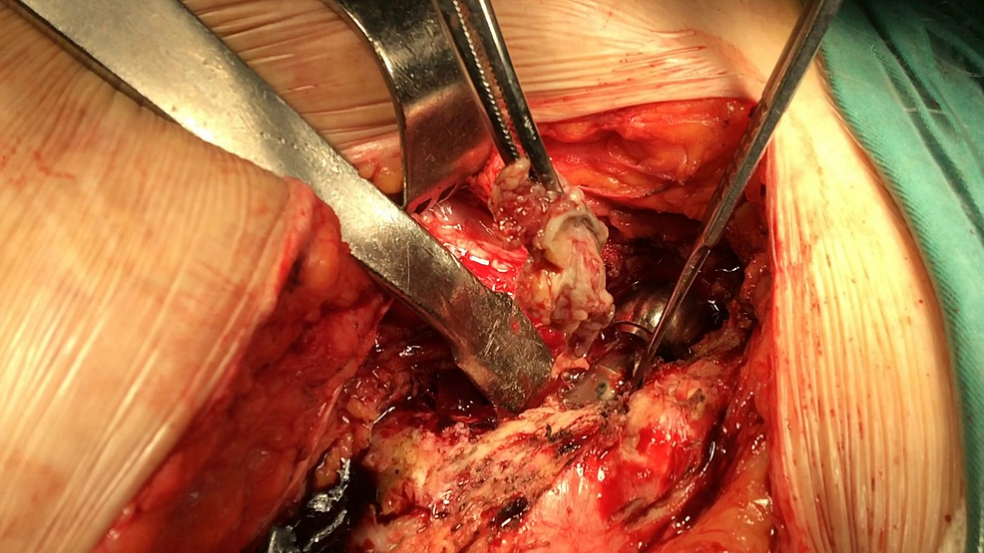
Intraoperative findings - soft tissue inflammation

Despite the obvious inflammation of soft tissue intraoperatively (Figure 2), *P. asaccharolyticus* was isolated in two out of six tissue samples and collected fluid using standard microbiological techniques. After sonication of removed implants with low-intensity ultrasound for the disintegration of biofilm, *P. asaccharolyticus* was the only microorganism isolated and cultured in the sonication fluid (Table 2). A six-week antibiotic-specific therapy with intravenous ceftriaxone and daptomycin was started immediately based on microbiological sensitivity results and was conducted under the close supervision of the Infection Control Department of the hospital. The six-week period was completed successfully without any adverse events. ESR and CRP were performed and were 55 mm/h and 3.1 mg/dL, respectively, still exceeding normal values. The patient was discharged home with the instructions to continue taking clindamycin orally for another month.

**Table 2:** Microbiology result after sonication of removed implants and antibiotic sensitivity MIC: minimum inhibitory concentration

Antibiogram of anaerobe P. asaccharolyticus isolated in sonication fluid of hip arthroplasty implants					
	MIC_90 _(μg/mL)	Susceptibility %		MIC_90 _(μg/mL)	Susceptibility %
Penicillin	0.047	98.4%	Doxycyclin		
Amoxillin/Clavulanate	0.047	100%	Chloramphenicol		
Piperacillin/Tazobactam	0.047	100%	Tetracycline		
Ceftriaxone	<0.002	100%	Clindamycin	0.094	97.37%
Meropenem	<0.002	100 %	Metronidazole	0.5	100%

After a two-month period free of any antibiotics, the 68-year-old patient returned for re-implantation of the prosthesis. On re-admission, her serum infection markers were normalized: erythrocyte sedimentation rate was 20 mm/h (normal: 0-20) and C-reactive protein was 0.31 mg/dL (normal: <0.5).

**Figure 3:**
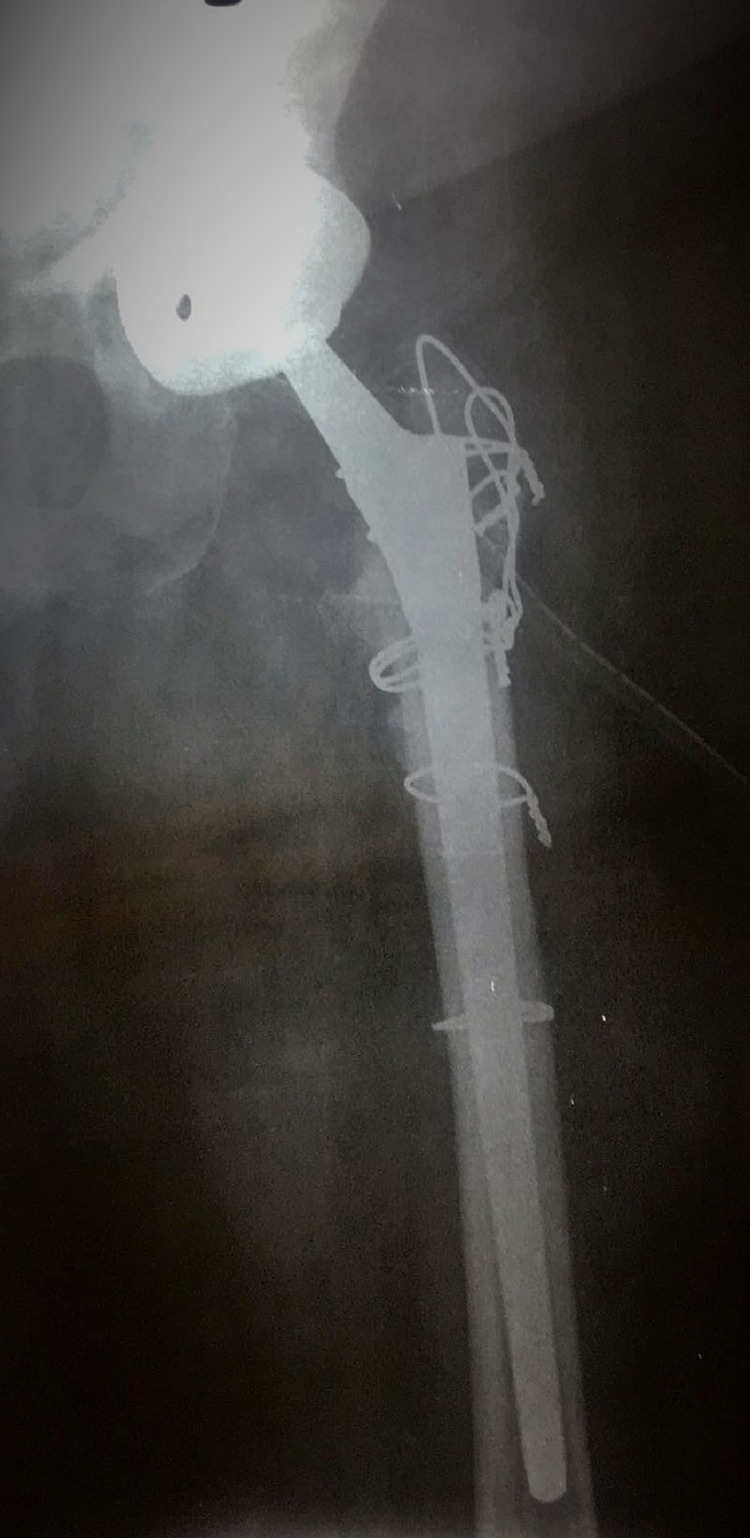
Patient’s postoperative X-ray

Spinal anesthesia and a posterior approach were used. Intraoperatively, soft tissues looked in good condition and the bone stock was adequate. Tissue samples were taken for cultures and histology. A continuum uncemented cup prosthesis (Continuum TM cup, Zimmer Biomet, Warsaw, IN, USA) with screws and an uncemented Wagner revision stem was used (Zimmer Biomet, Warsaw, IN, USA) (Figure 3). Post-operatively, she was treated with intravenous vancomycin till we had the results of the intra-operative cultures. All cultures were negative and the antibiotics were stopped on the fifth postoperative day. She was mobilized with partial weight bearing on the second post-op day and was discharged home on the fifth post-op day.

At her last follow-up, one year after her final hospital discharge, she was able to walk independently without pain or relevant symptoms in the affected hip. The radiographic evaluation was excellent, and the serum infection markers were normal.

## Discussion

Anaerobic microorganisms are responsible for only 3-6% of PJI [2], but they cause serious complications and poor outcomes for the patients. *P. asaccharolyticus* is a GPAC and is usually found in polymicrobial infections. In their study analyzing the colonization of chronic wounds in 60 patients, Choi et al. ranked Peptoniphilus among the most prevalent anaerobic pathogens [3].

Even so, there is not enough evidence about the frequency of *P. asaccharolyticus* in more serious infections, like periprosthetic joint infections, which take on more malignant aerobic microorganisms such as *Staphylococcus aureus*, Streptococcus spp., and Enterobacterales.

The polymicrobial nature of GPAC and the difficulty of culturing with standard microbiological techniques restricted the extensive study of these infections. Defining microbiological results can be delayed for up to two weeks due to the prolonged cultivation times needed for the isolation of the anaerobes while infection treatment has already started. Microbiological isolation techniques used to isolate and identify these rare and challenging anaerobic bacteria were not as routinely used in the past as they are now [4]. What is more, great changes in the classification system of anaerobe bacteria have been proposed and may confuse the bacterium-disease association [4].

Walter et al. [5] studied 61 cases of anaerobic bone and joint infections and reported that 82% of them involved polymicrobial infections. Similar studies reported that up to 46% of bone and joint infections are caused by more than one pathogen [6]. Brown et al. studied 15 cases of hematogenous infections caused by Peptoniphilus ssp. in patients with underlying diseases, including such as soft tissue, respiratory, and urinary tract infections, and stated that Peptoniphilus is a rare but significant cause of hematogenous infections [7]. These studies underline that *P. asaccharolyticus* is a common "teammate" in infections caused by multiple microorganisms, but its main role as a commensal, pathogen, or synergist is still a mystery. Verma et al. presented the only case of *P. asaccharolyticus*-associated serious monoinfection: a 55-year-old diabetic woman with hip septic arthritis and osteomyelitis [8].

Biofilm formation at the implant surface is a major factor in chronic periprosthetic infections and has a crucial role in the lack of positive cultures of periprosthetic soft tissue samples obtained intraoperatively. The synergistic effect between GPAC and more virulent pathogens like S. aureus and Pseudomonas spp. is a result of the biofilm formation, which increases the pathological impact of GPAC. Consequently, GPAC prefers to live and multiply when co-existing within a polymicrobial biofilm layer [3].

Sonication of removed implants with the use of low-intensity ultrasound for the disintegration of biofilm and the culture of the sonication fluid is an effective and more sensitive method for the diagnosis of PJI than conventional tissue cultures [9]. By comparing sonication to standard tissue cultures, Bozic and Ries studied 331 patients with PJI, including THR and TKR, and found that sonication fluid cultures have higher sensitivity and similar specificity (sensitivity of 60.8% and 78.5%, and specificity of 99.2% and 98.8%, respectively) [9]. Sonication fluid cultures were not detected by standard techniques and had significantly higher sensitivity (75% versus 45%) in patients who received antibiotic therapy prior to surgery.

## Conclusions

Our study and literature review highlight a different aspect of *P. asaccharolyticus* as a causative pathogen in serious mono-infections and induce new hypotheses that need further evaluation. Due to microbiological isolation, cultivation difficulties, and the superiority of aerobic bacteria in the polymicrobial spectrum, the frequency of *P. asaccharolyticus* infection in serious periprosthetic joint infection may be higher than previously assumed. Furthermore, the pathogenicity of *P. asaccharolyticus* as a primary causative pathogen in more serious infections may not have been evaluated properly, and more studies on this subject are still needed. The role of *P. asaccharolyticus* as the causative pathogen in mono-infection should be taken seriously. The present case report and review of the literature may represent a starting point for clinical and scientific investigation.

## References

[1] Tande AJ, Patel R (2014). Prosthetic joint infection. Clin Microbiol Rev.

[2] Dowd SE, Wolcott RD, Sun Y, McKeehan T, Smith E, Rhoads D (2008). Polymicrobial nature of chronic diabetic foot ulcer biofilm infections determined using bacterial tag encoded FLX amplicon pyrosequencing (bTEFAP). PLoS One.

[3] Choi Y, Banerjee A, McNish S (2019). Cooccurrence of anaerobes in human chronic wounds. Microb Ecol.

[4] Shenoy PA, Vishwanath S, Gawda A (2017). Anaerobic bacteria in clinical specimens - frequent, but a neglected lot: a five year Experience at a tertiary care hospital. J Clin Diagn Res.

[5] Walter G, Vernier M, Pinelli PO, Million M, Coulange M, Seng P, Stein A (2014). Bone and joint infections due to anaerobic bacteria: an analysis of 61 cases and review of the literature. Eur J Clin Microbiol Infect Dis.

[6] Brook I (2008). Microbiology and management of joint and bone infections due to anaerobic bacteria. J Orthop Sci.

[7] Brown K, Church D, Lynch T, Gregson D (2014). Bloodstream infections due to Peptoniphilus spp.: report of 15 cases. Clin Microbiol Infect.

[8] Verma R, Morrad S, Wirtz JJ (2017). Peptoniphilus asaccharolyticus-associated septic arthritis and osteomyelitis in a woman with osteoarthritis and diabetes mellitus. BMJ Case Rep.

[9] Bozic KJ, Ries MD (2005). The impact of infection after total hip arthroplasty on hospital and surgeon resource utilization. J Bone Joint Surg Am.

